# A spatiotemporal deep learning framework for detecting unusual human activity in surveillance videos

**DOI:** 10.1371/journal.pone.0355690

**Published:** 2026-09-23

**Authors:** Manoj Kumar, Mantosh Biswas, Anoop Kumar Patel, Abhay Kumar, Kumar Abhishek, Ahamed Shafeeq B. M.

**Affiliations:** 1 School of Computer Science Engineering & Technology, Bennett University, Greater Noida, India; 2 Department of Computer Science, North Campus, University of Delhi, Delhi, India; 3 National Institute of Technology, Kurukshetra, Haryana, India; 4 Department of Computer Science and Engineering, NIT Patna, Patna, Bihar, India; 5 Manipal Institute of Technology, Manipal Academy of Higher Education, Manipal, India; Shanghai Maritime University, CHINA

## Abstract

With smart gadgets and computers everywhere, real-time video monitoring is now necessary to keep people secure and lower the chance of unexpected human actions. However, current surveillance systems have a lot of trouble effectively spotting unusual actions in real life. Most of the advanced anomaly detection algorithms are trained and tested on synthetic or simulated video datasets. These datasets frequently do not show how complicated and different real-life settings can be. Because of this, their usefulness and capacity to be used in real life are still restricted. We propose a transfer learning-based hybrid deep framework for finding unusual activity in real-world surveillance. We test the model using raw video streams from a subset of the UCF-Crime dataset that shows actual situations of people doing things. First, a pre-trained DenseNet-201, a convolutional neural network, extracts spatial data from video frames that have already been processed. A Bidirectional Long Short-Term Memory (Bi-LSTM) network is used to describe temporal relationships between video sequences in a useful way. The spatial data and the Bi-LSTM’s temporal representations are then integrated to make anomaly identification more precise and dependable. This integrated spatiotemporal architecture identifies abnormal actions of human beings in challenging monitoring situations. The model that was recommended performs better than the state of art methods. It gets 95.04% accuracy on the UCF50 dataset and 62.04% on the untrimmed UCF-Crime dataset. These results suggest that the model performs effective in real-life scenarios.

## 1. Introduction

Activities, like keeping an eye on the environment, managing traffic, and solving crimes, require smart city management to be monitored. People are more worried about safety in cities; thus, more surveillance recordings are being used in public places to monitor people and prevent crimes. Abnormal action identification is difficult as there is a large variation in human actions. Abnormal activity identification is the technique of finding human actions that do not seem right or acceptable in society from videos. It means figuring out whether the current video frames show anything unusual happening or not. Unusual human actions are things that happen that are not regular, anticipated, or conventional.

Recognizing abnormal human actions in videos is a challenging field, gaining significant attention from researchers due to its relevance as a pressing social issue. Applications of human action recognition in videos enhance surveillance systems, intelligent scene modeling, video annotation, and retrieval. Despite challenges related to privacy and certain limitations, video surveillance plays a vital role in improving security, safety, and trust. Reviewing surveillance recordings over an extended time is both time-consuming and difficult. Additionally, the video contains a very short period of anomalous events out of the whole span of the video, which may be onerous and inefficient in manual processing. Consequently, there is a growing need for an automated system to detect abnormal activity.

Traditional approaches often employ trajectory-based anomaly detection, where a video is considered anomalous if the objects of interest deviate from predefined normal trajectories [1,2]. These solutions need extensive monitoring, which is unsuitable for surveillance footage. Furthermore, when it is applied to a different area, these typical procedures are unsuccessful. They are unsuitable for adapting to a new environment and fail to identify abnormal activity. Since deep learning algorithms have been so successful in computer vision, current studies have focused on applying deep learning methods for the detection of aberrant human activities. Deep learning algorithms can extract two types of information: spatial characteristics and temporal features. CNN and other existing deep learning models [3–6] have been shown to exhibit optimum performance for spatial information. Several methods for temporal features have been proposed in the recent decade, and they all come with their own set of benefits and drawbacks.

Automating Threat Recognition System [7] is a real-time surveillance application that uses deep learning algorithms to aid in the rapid identification of suspicious activities while cutting down on wasted time and effort. Real-time, automated detection of aggressive and violent behavior is being developed in the hopes of eliminating false positives. The Deep Learning model – CNN will be used to detect and categorize extreme motion in the video. Automatic detection of anomalies is a difficult task due to the absence of annotated anomalous events, low-resolution videos, and significant intra/inter-class variations between the number of classes.

To learn local features and classify abnormal activities, a feed-forward deep auto-encoder-based fully CNN technique is utilized [8]. The network is given normal videos during the training phase, and features are retrieved. During the testing phase, videos with out-of-the-ordinary events are predicted to have a high reconstruction error. For quantitative analysis, they have provisional segmented the anomalous activities in the video. This model is not tailored to a specific dataset but broad enough to pick up commonalities in several types of data. The most regular frame is synthesized from the test video by collecting the pixels with the highest regularity score and running them through a convolutional autoencoder and an autoencoder on better trajectories. They search for local minima in the regularity score time series to identify out-of-the-ordinary occurrences. On the other hand, these local minima were quite loud, and not all were relevant local minima. The proposed method [9], which employs a fully connected convolutional feed-forward auto-encoder, correctly learns these patterns; however, it also generates large anomaly scores, even for previously learned normal patterns.

This research aims to exploit the spatiotemporal characteristics of complex abnormal human activity datasets and correctly distinguish their classes. In the proposed CNN-RNN framework, surveillance video frame features are obtained using a pre-trained CNN, while an RNN model is employed to learn their temporal information for anomaly detection. The proposed framework is capable and strong to compare with present techniques, and it achieves state-of-the-art performance on large-scale, complex abnormal datasets of human activity for multi-class categorization. The following is a key contribution of our work:

- We extract the features of the frame using DenseNet-201, a pre-train network that utilizes dense connectivity to ensure efficient feature reuse and mitigate vanishing gradient problems.
- We then feed these features into the sequential model based on Bi-LSTM that employs bidirectional processing to capture both past and future context, enhancing understanding of temporal dynamics.
- Integration of Transfer Learning influences pre-trained models, i.e., **DenseNet-201**, on large datasets and fine-tunes on specific anomaly detection tasks.
- We compare the efficacy of our model to current best practices.

The paper contains 5 sections explaining the architecture and implementation of deep learning for abnormal activity recognition. Section 1 is the introduction that briefly describes the basic motivation and basics of the problems. Section 2 talks about the literature survey, and Section 3 talks about the proposed design, while Section 4 represents the implementation of the project and discusses all the features and functionalities. The conclusion and future work are described in Section 5.

## 2. Literature survey

Over the last few years, the high growth rate of smart video surveillance systems has substantially changed the concept of safety in the masses, smart cities, and self-monitoring systems. As the amount of video data available to researchers has increased, with the advent of artificial intelligence, there has been an increasing interest in real-time surveillance footage in identifying abnormal or suspicious activity. Early works were mostly based on handcrafted features and classical machine vision techniques. However, subsequent ones used motion analysis, audio-visual fusion, and learned complex behavioral patterns using deep neural architectures. Despite ongoing developments, the detection of abnormal activities has become a difficult task because of the change in the dynamics of scenes, occlusions, and crowded scenes, the limited availability of labeled anomaly data, and the change in the definition of abnormality in different situations. To give a better view of the development of the research field, the available literature has been systematically examined and classified according to methodological advancement. The research mentioned in the previous literature can be classified into three broad categories: Conventional (Handcrafted/ Rule-based) Approaches, Motion-based Methods, and Deep Learning-based Methods.

### 2.1. Conventional approach

In this approach, the features are extracted by feature engineering, and a classifier is used to classify whether the weather video stream is normal or abnormal. Various studies have been conducted using this approach. Tian et al. [10] were able to approximate activity recognition in a congested environment. The 2D Harris corner detector [11] was utilized to identify the point of interest located at the high intensity point in the MHI template. HOG additionally represents spatiotemporal characteristics, while actions are categorized by the Gaussian Mixture Model (GMM) model [12]. The Space-Time 3D shape model of the MEI template, introduced by Blank et al. [13], utilizes binary silhouettes to surpass the performance of prior methods employed in human action detection. Their methodology surpasses the need for video alignment and has the potential to be implemented in practical situations. Work proposed by Weinland et al. [14] that uses spatiotemporal features to identify abnormal human activities in videos that are view-invariant. The authors [15] introduced the BOF (STIP) methodology, which involved the expansion of the 2D Harris detector to incorporate a 3D corner detector. The STIP features-based representation for pose estimation under conditions of obstructed background and view variation has demonstrated outstanding performance. Nevertheless, their methodology is susceptible to camera motion, specifically camera glitches. In order to expand the 2D Hessian detector to 3D, Willems et al. [16] proposed using the second derivatives of the corner detector to focus on specific actions. Spatial filtering approaches were initially proposed by Dollar et al. [17] as a means to identify and characterize behavior in video sequences. Klaser et al. [18] introduced an action descriptor that uses a histogram of oriented spatiotemporal information in three dimensions. Descriptors based on HOG features have been expanded and generalized to encompass 3D spatiotemporal volumes, enabling the detection of action in videos. Laptev et al. [19] introduced a new approach called “Histogram of Optical Flow (HOF)-based spatiotemporal descriptor” to improve the training of an action classifier. A human pose descriptor was devised by Dalal et al. [20] utilizing the Histogram of Oriented Gradients (HOG) to identify motion in dynamic environmental conditions. Zhu et al. [21] proposed a max-pooling sparse coding framework using features extracted from dense spatiotemporal feature cuboids.

Overall, these approaches, which operate on handcrafted spatiotemporal descriptors, illustrate the effectiveness of these methods in reflecting motion patterns to detect abnormal activities. Nevertheless, their operation is frequently constrained by camera motion sensitivity, changing perspective, and real-life issues, which are driving the transition to more robust deep-learning-based techniques.

### 2.2. Motion-based approach

In this approach, the extracted features are basically the motion pattern of humans in the video stream. Bobick and Davis [22] utilized motion history images (MHI) and motion energy images (MEI) temporal templates to extract the motion feature from video sequences to identify human action against congested backgrounds. Their attention has been drawn to particular human motion activities, and motion is evaluated gradually. Laptev et al. [19] introduced a new approach called “Histogram of Optical Flow (HOF)-based spatiotemporal descriptor” to improve the training of an action classifier. A human pose descriptor was devised by Dalal et al. [20] utilizing the Histogram of Oriented Gradients (HOG) to identify motion in dynamic environmental conditions. Zhu et al. [21] proposed a max-pooling sparse coding framework using features extracted from dense spatiotemporal feature cuboids. A HOG3D [23] descriptor was utilized to extract the spatio-temporal features. Every extracted feature is sparsely encoded using a dictionary that has been pre-trained, and max pooling is performed on the complete sparse code for every video. For video classification and behavior analysis, Guha and Ward [24] introduced a sparse model for human action recognition that is based on a learned lexicon. The optical flow[manoj] was being utilized to extract the specific pattern in the video. Nguyen et al. [25] propose the utilization of a Gaussian descriptor to depict action and pose, which is constructed from high-order statistics of local features distributed across two levels. A multi-modal fusion method for shared feature learning was introduced by Kong et al. [26] to depict the local dynamics of each joint action class. Action classification is accomplished utilizing a maximum-margin framework. In their study, Raman and Mayank [27] introduced a two-level hierarchical model designed for activity recognition with the assistance of depth sequences and skeleton coordinates.

### 2.3. Deep learning-based approach

The methods of deep learning have immensely contributed to the area of anomaly detection and activity recognition by making it possible to learn features hierarchically automatically on complex and high-dimensional data. These methods adopt convolutional, recurrent, and attention-based networks to obtain complex spatial-temporal relationships, making them more accurate and robust in detection. Deep learning techniques like CNN and bidirectional LSTMs are also used [28]. These models are used to learn spatial and temporal characteristics and were trained on both unusual and common occurrences. The proposed method has the benefit of being semi-supervised and is also used to learn and classify local features using fully connected convolutional feed-forward auto-encoder-based techniques [29]. During the training phase, the network is shown regular movies, and features are extracted. Although the proposed method is good at picking up on familiar patterns, it tends to provide high anomaly ratings even for fresh examples of the norm. The VGG-16 model [30] is implemented, with CNNs for feature extraction and RNNs with Bi-LSTM for finding latent temporal or sequential patterns in the input data. Anomaly and normal CCTV footage from the real world were analyzed in study [31] to handle the complexity of dealing with realistic anomalies. A universal model of anomaly detection was also learned, and this was done with the help of two different neural networks. The recent researchers [32] on the topic of crowd analysis had highlighted the need to comprehend the motion patterns, sources, and sinks to be able to interpret the scene correctly. Khan et al. [33] presented a particle-advection and optical-flow-based method and used it to compute dense trajectories and detect prevailing pedestrian flows. Previous tracking-based solutions were not successful in dense scenes because of occlusions and poor reliability of features. Such developments demonstrate the importance of powerful motion models in video surveillance and anomaly activity detection.

Tu et al. [34] present a graph frequency analysis model of hyperspectral anomaly detection, which combines spectral features with the graph structure with a beta distribution-based adaptive graph wavelet space. The method exploited the high-frequency energy variation under anomaly to produce a high-quality detection performance using an entropy-based frequency localization strategy across various real HSI datasets. Qiao et al. [35] proposed a Multiheaded Attention Self-supervised (MAS) representation model of industrial sensor anomaly detection based on contrastive learning. This sensor sequence-specific architecture performed well on a realistic dataset of a water circulation system. According to Tu. et al. [36] Multi-scale Autoencoder Suppression Strategy (MASS) should be applied in hyperspectral anomaly detection, which enhanced the reconstruction of the background with the help of multi-scale feature extraction with convolutional and Transformer networks. Bukht et al. [37] came up with a human activity recognition system, which was based on the use of HSI color transformation, Gaussian filtering, silhouette extraction, feature extraction through BRISK and SIFT, and feature selection as being done with the Gray Wolf optimizer. The chosen features are grouped with the help of an Extended Kalman Filter with a recognition rate of 88% on the SUB-Interaction dataset and 86% on the HMDB51 dataset.

Amid remarkable advances made in the conventional, motion-based, and deep learning paradigms, the existing abnormal activity detection approaches still have serious limitations, limiting their application in real-life surveillance. Motion-based and handcrafted methods usually have issues with the dynamic scene variations, occlusions, and motion of the camera, and therefore cannot be as robust as they could be in uncontrolled situations. Even more powerful are deep learning models that are often based on synthetic or highly curated benchmark data sets, which do not reflect the complexity and unpredictability of real surveillance footage. Weak long-range temporal modeling is also common to many of the existing architectures, and it is therefore less capable of representing slow-evolving or context-specific anomalies. In addition, the computational complexity of 3D CNNs, hybrid networks, and multi-stream networks makes them less feasible in real-time applications, particularly in large-scale or resource-limited surveillance. All these problems point to the necessity of an efficient, context-aware, and temporally resistant framework that could work dependably on video streams in the real world. Out of these constraints, we suggest a new approach that will produce better temporal insights, less computational complexity, and better real-time response to practical abnormal human activity detection.

## 3. Proposed model

Detection of video anomalies demands the ability to jointly model a spatial and a temporal component; detection in one of the two is relatively easy, whereas jointly modeling to a reliable and real-time level is difficult. Our solution thus combines deep CNN-based spatial descriptors per-frame with a Bi-LSTM-based temporal aggregation and combines the two descriptions to make results on anomaly detection. In the case of spatial encoding, we apply DenseNet-201 due to several technical reasons. The dense pattern of connectivity of DenseNet provides reuse of features and better gradient flow, offering learning of fine-grained low-level and mid-level visual features that are useful for subtle abnormal behavior. DenseNets perform equally to very deep residual networks with a significantly smaller number of parameters, and with less feature redundancy, leading to a smaller memory footprint and inference cost, which is important in pipelines with near-real-time requirements. Transfer learning is further enhanced by the tight feature propagation, whereby pretrained DenseNet-201 weights are rich multi-scale features and thus need less fine-tuning to make them fit surveillance images, accelerating the convergence and enhancing their ability to generalize to novel camera perspectives or lighting conditions. To limit the latency and the usage of the GPU memory, we introduce chunk-based processing to capture the temporal context. A fixed frame rate is used to sample incoming video in small overlapping chunks. Frames of a chunk are preprocessed and then fed to DenseNet-201 until the global average-pooling layer; we pull out the per-frame feature vectors instead of the final output. The vectors in one chunk are stacked in time order to give a sequence. These sequences are then processed by the Bi-LSTM, which transforms spatial features into context-aware temporal embeddings by spatial–temporal fusion, performed through a temporal feature aggregation. The outputs from forward and backward LSTM passes are concatenated to form a bidirectional spatiotemporal representation. Several advantages have been offered by using chunked sequences: sequence length is limited, the Bi-LSTM models medium-range temporal dependencies without memory or gradient explosion; overlapping chunks make detection boundaries smoother and better localize events in time; it can be run in batches of large numbers of short sequences allowing it to be run on the GPU, and it can be run online since each chunk can be evaluated as soon as it has been formed (low latency) but they still offer the benefits of providing temporal localization. In practice, we freeze the initial blocks of the DenseNet and fine-tune it later with a reduced learning rate, extract 1,920-dim framewise vectors, use LayerNorm/dropout to stabilize training in the LSTM, and mask variable-length chunks. Lastly, the Bi-LSTM scores of anomalies are merged across overlapping chunks to generate strong real-time alerts and event classification. The model will be used to classify whether a video segment contains anomalous activity.

### 3.1. System architecture of the proposed model

**Fig 1:**
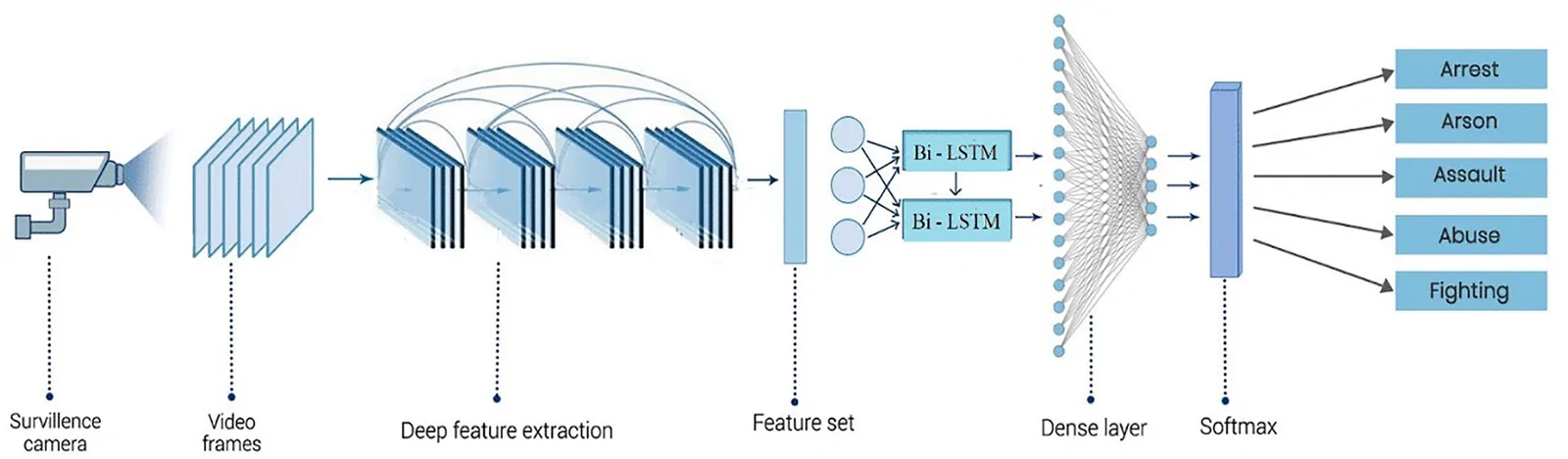
System Architecture.

The Abnormal Activity Recognition Model Architecture is presented in Fig 1, and the subsequent section explores each stage:

**Framing:** The initial step in the proposed approach is to extract frames from the collected CCTV recordings. These frames are preprocessed to adjust and resize images to the format required by the pre-train model DenseNet-201.These frames are converted to size 224 X 224 for processing. This resizing enables effective transfer learning while maintaining computational efficiency for real-time processing.

**Deep feature extraction:** In the next step, we use a pre-trained DenseNet-201 as a feature extractor for abnormal activity recognition. DenseNet-201, a deep CNN architecture, consists of multiple dense blocks, transition layers, and a classification layer. Each dense block includes several dense layers, where each dense layer contains multiple convolutional layers with feature concatenation. This connectivity design, in which each layer is associated with the next layer in a feed-forward manner, improves information flow and gradient propagation, successfully addressing the vanishing gradient problem. This results in improved feature extraction, especially for complex spatial structures.

A 201-layer convolutional neural network called DenseNet-201 is based on dense connectivity, whereby every layer gets the feature maps of the previous layers, allowing the reuse of features efficiently and strong gradient flow. The architecture consists of an initial 7x7 convolution, followed by a max-pooling layer, and four mainly closely connected blocks consisting of 6, 12, 48, and 32 dense layers, respectively. Each dense block has a block composed of batch normalization, ReLU activation, 1x1 bottleneck convolution, and 3x3 convolution, with its output being added to the prior outputs of the block. The three of these transition layers compress the channels with 1x1 convolutions and spatial down-sampling via average pooling. The spatial information is then pooled with a global average layer after the last dense block to a small pool of 1920-dimensional feature vector that we use as a spatial descriptor in our model. This architecture, which utilizes a deep feature hierarchy, an effective usage of parameters, and has high multi-scale representation ability, makes DenseNet-201 especially effective at extracting subtle spatial cues in video frames in order to identify abnormal activities. DenseNet-201’s dense blocks are composed of several dense layers, each comprising a series of convolutional layers with the same number of filters and kernel sizes, followed by feature concatenation. Transition layers are made up of a convolutional layer with 64 filters and a max pooling layer. It makes the spatial dimensions and the number of feature maps smaller. This design makes it easier to reuse features since each layer may access features from all the layers that came before it. This cuts down on the number of factors and makes it easier to get more varied and rich characteristics, which are very important for telling the difference between typical and unusual behaviors.

The input layer may take an RGB picture that is 224 by 224 by 3 pixels. The first layer, which is a convolutional layer with 64 filters of size 7x7 and a stride of 2, makes the feature map smaller in space. By putting the output into a max pooling layer with a pooling size of 3x3 and a stride of 2, the spatial dimensions are even less.

The result of using DenseNet-201 to extract features from the UCF Crime and UCF 50 datasets includes the features from the data it was trained on. This makes it possible to recognize aberrant behavior quite well. This design makes feature reuse and gradient propagation easier, which leads to improved feature extraction and better detection of unusual actions in video surveillance.

**Fig 2:**
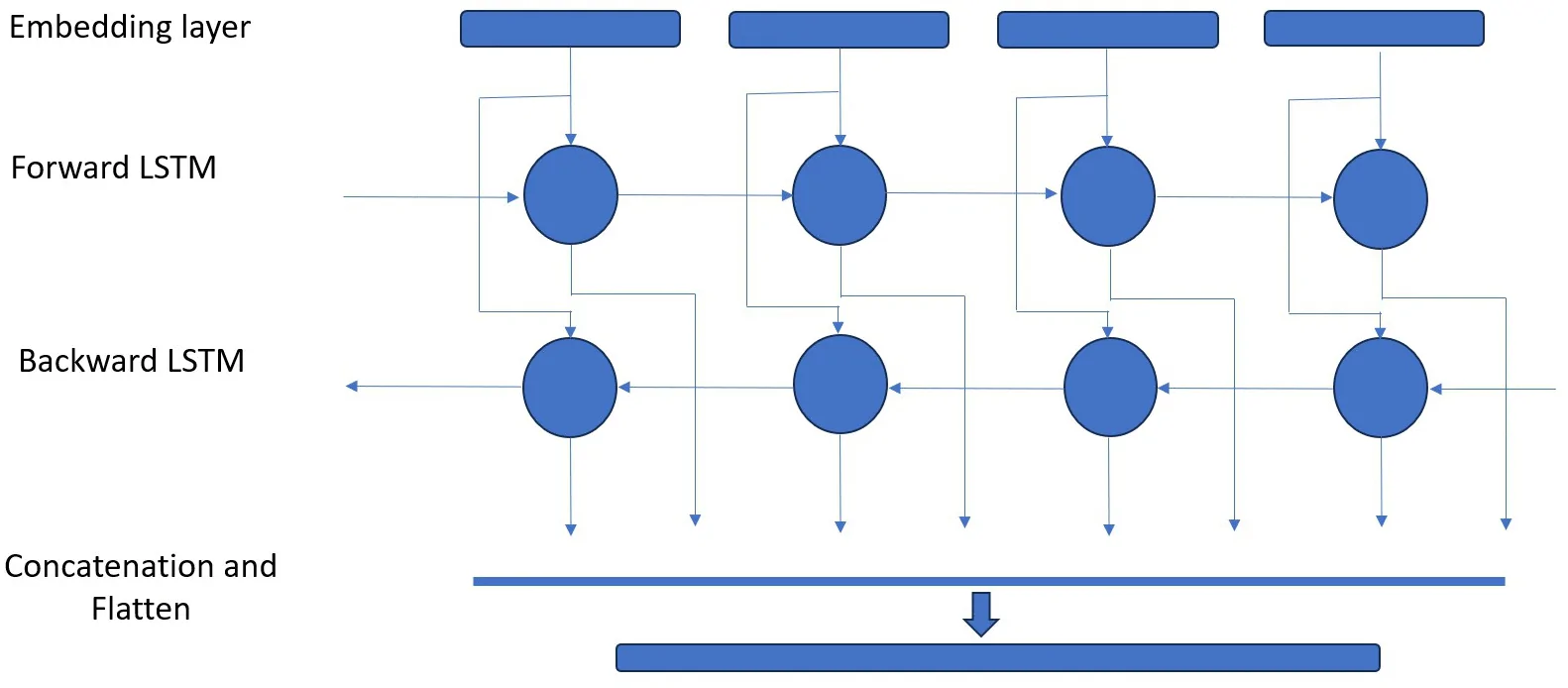
Bi-LSTM Architecture.

**Training the model:** We are utilizing a Bi-LSTM to analyze the sequences of data once we have extracted the features. The LSTM portion of Bi-LSTM is a form of RNN that solves the issue of vanishing gradients by letting error gradients spread out over extended periods of time. The LSTM network has memory cells, input layers, output gates, and forget gates that control how information moves through the network. The Bidirectional portion of Bi-LSTM denotes that the input sequence is processed in both forward and backward directions, with distinct hidden states and output layers for each direction, as seen in Fig 2. The forward LSTM looks at the input video from start to finish, whereas the reverse LSTM looks at it from end to start. This lets the network remember both the past and the future dependencies of the input sequence. This is especially helpful for tasks like activity identification, where the meaning of an action may be affected by both the words that come before and after it. The Bi-LSTM network gives back an array of hidden states as its output. It may utilize these concealed states to label, classify, or create sequences. LSTM networks may learn and find long-term dependencies. The cell state is the most important part of LSTMs since it lets information move quickly. Gates are devices that only let certain types of information through. It uses a pointwise multiplication technique and a sigmoid neural network layer, put together to form the forward and reverse LSTMs.

An LSTM incorporates three gates to safeguard and control the cell state: Forget Gate (f_t_), Input Gate (i_t_), and Output Gate (O_t_). Additionally, it maintains a cell state (C_t_) and a hidden state(h_t_). The forget gate layer is used to ascertain if certain info need to be disregarded or not using equation (1).


$$\begin{array}{c} f_{t}=\sigma \left(w_{f}\;.\left[h_{t-\text{1}},x_{t}\right]+b_{f}\right) \end{array}$$ (1)

Where $w_{f}$, bf, and σ are the weight matrix, bias vector, and sigmoid activation function, respectively. $h_{t-\text{1}}$is previous hidden state, and $x_{t}$ represents the current input for the forget gate.

The input gate first runs $h_{t-\text{1}}\;$and $x_{t}$ through the sigmoid function to decide which values to update. Then, it creates a vector of new candidate values ${\underset{―}{C}}_{t}\;$to be added to the cell state using the tanh function. This is illustrated by equations (2) and (3).


$$\begin{array}{c} i_{t}=\sigma \left(w_{i}\;.\left[h_{t-\text{1}},x_{t}\right]+b_{i}\right) \end{array}$$ (2)

$$\begin{array}{c} {\underset{―}{C}}_{t}=\;tanh\left(w_{c}\;.\left[h_{t-\text{1}},x_{t}\right]+b_{c}\right) \end{array}$$ (3)

Where $w_{i}$ is the weight matrix, $b_{i}$ is the bias vector, σ:sigmoid activation function, $h_{t-\text{1}}$is previous hidden state, and $x_{t}$ is the current input for the input gate. $w_{c}$ and $b_{c}$ are the weight matrix and bias for the candidate cell state, respectively.


$$\begin{array}{c} C_{t}=f_{t}\circ c_{t-\text{1}}+i_{t}\circ {\underset{―}{C}}_{t} \end{array}$$ (4)

The new cell state is computed by combining the old cell state, scaled by the forget gate $f_{t}$, and the candidate values, scaled by the input gate $i_{t}$, using equation (4).

The output gate takes $h_{t-\text{1}}\;and\;x_{t}$ and runs them through the sigmoid function to decide which parts of the cell state should be output. The cell state is then passed through the tanh function to push the values to be between −1 and 1 and multiplied by the output of the sigmoid gate to get the final hidden state, $h_{t}\;$. This is described by equations (5) and (6).


$$\begin{array}{c} o_{t}=\sigma \left(w_{o}\;.\left[h_{t-\text{1}},x_{t}\right]+b_{o}\right) \end{array}$$ (5)

$$\begin{array}{c} h_{t}=\;o_{t}\circ tanhtanh\;\left(C_{t}\right) \end{array}$$ (6)

The LSTM network combines the input, forget, and output gates to control the information flow, allowing it to hold information over long periods and avoid issues like vanishing gradients.

To handle the challenge of using Bi-LSTM for live video streams, where future frames are not available, we employ Chunk-Based Processing strategies that adapt Bi-LSTM to work effectively in real-time scenarios. In this strategy, we divided the incoming video into overlapping chunks, for example, 20 frames with 50% overlapping. This overlapping ensures the temporal dependencies across chunks are not lost after applying the Bi-LSTM on every chunk. As new frames arrive, shift the window and process the next chunk to ensure the model is continuously updated with fresh data. It may introduce minor delays.

Chunk-Based Processing Algorithm

Input: Video V with frames F1, F2, …, Fn

Parameters: chunkSize (C), overlapRatio (r),DenseNet-201 model D,Bi-LSTM model L, stride S = C * (1 – r)

***Algorithm***

  *Sample frames at fixed fps*

  *Normalize each frame using DenseNet preprocessing.*

   *featurSequenceList ← empty list*

   *chunkStart ← 1*

   *While chunkStart ≤ n:*

    *Step 1: Extract chunk frames*

     *chunkFrames ← F[chunkStart: chunkStart + C – 1]*

     *If length(chunkFrames) < C:*

     *Break;*

    *Step 2: Extract spatial features using DenseNet-201*

     *chunkFeatures ← empty list*

     *For each frame f in chunkFrames:*

     *featureVector ← D(f)*

     *chunkFeatures.append(featureVector)*

    *Step 3: Convert list to sequence tensor*

     *sequenceTensor ← Stack(chunkFeatures)*

     *shape = (C, featureDim)*

    *Step 4: Temporal modeling using Bi-LSTM*

      *lstmOutput ← L(sequenceTensor)*

    *Step 5: Chunk-level representation*

     *chunkEmbedding ← Aggregate(lstmOutput)*

    *Step 6: Classification or anomaly scoring*

     *chunkScore ← Classifier(chunkEmbedding)*

   *Store result*

     *featureSequenceList.append(chunkScore)*

     *chunkStart ← chunkStart + S*

     *Combine chunk scores (mean, max, or time-weighted fusion)*

**Computational Complexity and Real-Time Analysis:**

Computational complexity can be analyzed in two parts: Spatial Feature Extraction (DenseNet-201) and Temporal Modeling (Bi-LSTM).

**Spatial Feature Extraction (DenseNet-201**): Let, = number of frames per chunk, = number of convolutional parameters, = feature dimension (1920 after global pooling)

The approximate per-frame convolutional complexity is:

However, the practical computational cost is reduced due to:

- Global average pooling (removes heavy FC layers)
- Freezing early dense blocks during inference
- Reuse of pretrained weights (no full backpropagation in deployment)
- Input resizing to 224 × 224 (fixed computational grid)

For chunk size:

**Temporal Modeling (Bi-LSTM):** Because feature extraction is parallelizable across frames on the GPU, the effective runtime scales efficiently.

For chunk size and hidden dimension:

Since it is fixed (e.g., 20), this becomes constant time per chunk:

Thus, total per-chunk complexity:

Because it is small and fixed, the system achieves predictable latency.

**Model Interpretability and Explainability:** Real-world deployment of abnormal activity detection systems needs interpretability as a crucial factor because automated decisions should be clear and comprehensible to security analysts. The proposed spatiotemporal framework will support interpretability at both the spatial and temporal levels. On the spatial scale, DenseNet-201 is used to obtain hierarchical visual representations of input frames with densely connected convolutional networks. The medium convolutional feature maps retain discriminative spatial information like object posture, interaction patterns, and the context of the scene. Based on the intensities of activities in the deeper convolutional layers, one can determine which parts of a frame contribute most to the prediction of abnormality. Due to dense connectivity, which facilitates the reuse of features and multi-scale representation, subtle abnormal visual patterns can be followed through the layer-wise feature activations.

Bidirectional dependencies of frame sequences within a chunk are captured in a Bi-LSTM network at the temporal level. The concealed state change records motion development and contextual transformation with time. With the help of measuring the magnitude of temporal activation and the change in hidden states with the frames, the impact of a particular motion segment on the final anomaly score can be more comprehensible. The chunk-based overlapping scheme also provides the opportunity to smooth the time, so that the localization of the anomalous chunks can be systematically provided.

Even though the current work is mainly devoted to the enhancement of detection accuracy and temporal robustness, more sophisticated explainability capabilities, including gradient-based activation maps and time attention visualization, can be incorporated into the current architecture to produce frame-level heatmaps and event-level explanations. Such improvements would further boost the transparency and confidence of the analysts in automated surveillance systems.

**SoftMax:** SoftMax is a common activation function used in neural network output layers for classification tasks. The aberrant human activity is classified using the SoftMax activation function. SoftMax accepts a vector of real-valued numbers as input and returns a vector of probabilities that sum to 1. The input to the SoftMax function is a vector of real-valued numbers z = [z1, z2,..., zK], where K is the number of classes. The SoftMax function first exponentiates each element in the input vector, i.e., it calculates the exponential function of each element: $e^{z_{\text{1}}},\;{\;e}^{z_{\text{2}}},\;e^{z_{\text{3}}}\;\ldots .\;e^{z_{k}}$. These values are then normalized so that they sum up to 1. The output of the SoftMax function is a vector of probabilities that represent the likelihood of the input belonging to each of the K classes. For example, if K = 5 and the output of the SoftMax function is [0.5, 0.1, 0.1, 0.2,0.1], it means that the input is predicted to belong to the first class with a probability of 0.5, the second class with a probability of 0.1, the third class with a probability of 0.1, the fourth class with a probability of 0.2, and the fifth class with a probability of 0.1.

### 3.2. Hyperparameter tuning

Hyperparameter tuning refers to the process of determining which values for a model’s hyperparameters will yield the best results. The training session consists of multiple trials combined into one. Every test is a full iteration of your training app with carefully chosen hyperparameter values that are inside the bounds you establish. After finishing this procedure, you will obtain a list of optimal values for the model’s hyperparameters.

One of the hyperparameters of the optimizer is the learning rate. The size of the step for a model must be taken to minimize the loss function, which is set by the learning rate. If the learning rate is increased, the model will learn more rapidly, but it may not get any closer to the least loss function. The possibility of discovering a minimum loss function improves with a reduced learning rate. In exchange for a slower learning rate, you’ll need more epochs, or time and storage space. Learning speeds up with smaller batches, but there’s more diversity in validation dataset correctness. While a bigger batch size slows down the learning process, it also reduces the variability in the correctness of the validation dataset.

Each time the neural network model is applied to a dataset, this process is called an epoch. In a neural network, the training dataset is only sent forward and backward once per epoch. When the number of epochs is too small, underfitting occurs because the neural network has not learned enough. One needs numerous epochs, or iterations, through the training dataset. Overfitting happens when a model can predict previous data but not new data. You may get the best outcome by changing the total number of epochs.

## 4. Implementation and results discussion

We did our study using the Google Colab research tool. This platform is centered on documents and makes it easy and quick for people to work together on code, data analysis, and research. The platform is the best place to execute Python code since it has pre-installed libraries, supports GPU/TPU, and has cloud-based storage that can be accessed from any device connected to the internet.

We used a local development environment with a quad-core Intel Core i7 Skylake CPU, which is known for its high performance and ability to handle heavy workloads. The system included 16 GB of RAM, which made sure that it could handle enormous datasets and complicated tasks without running out of memory.

### 4.1. Dataset

We used two very difficult datasets for people working together to move: UCF-50 and Sub-UCF Crime.

**UCF-crime:** The UCF crime [38] dataset is a complete collection of recorded criminal events that includes a wide variety of crimes, such as arson, assault, vandalism, and fighting. It gives us important information on the safety and security environment. Law enforcement, administrators, and researchers may use this information to find high-risk regions, understand crime trends, and put in place effective preventative initiatives to keep people safe and healthy.

**UCF50:** The UCF-50 dataset [39] is a standard dataset that is often used in computer vision and object recognition. There are 50 activity categories, and each one has real films of people doing different things, including walking, biking, diving, and more. This dataset is a regular way to test how well algorithms and models that are meant to identify and categorize human behaviors in video footage work. The UCF-50 dataset is still an important tool for academics who want to create action recognition systems that are both accurate and strong. This is because it has high-quality video footage and a wide range of activities.

### 4.2. Implementation details

To strike a balance between simplicity and performance, our network architecture samples 200 frames from each video at regular intervals. To preserve the semantic coherence of video sequences, this paper does not selectively remove the starting T frames. Instead, it collects frames at intervals of over a movie with N frames. If a video sequence is fewer than 200 frames long, the last frame will be repeated until the sequence is long enough. At last, we pick the subsequence S containing T frames by equation (7).


$$\begin{array}{c} Tnew=\left\{\text{0},\;\text{1}*\frac{N}{T},\text{2}*\frac{N}{T},\text{3}*\frac{N}{T},\text{4}*\frac{N}{T},\ldots n*\frac{N}{T}\right\} \end{array}$$ (7)

DenseNet-201 is used as the foundation for the feature extraction module, although only a single fully connected layer is maintained. Its intricate structure, which is utilized in our extraction module, is shown in Fig 1. Each frame is enlarged from its original dimensions to 224 by 224. Each frame used for the training of our model is modified by preprocessing, i.e., cropping, vertical flipping, horizontal flipping, adjusting the lighting, and introducing random noise. When the settings of the deep features extraction module (DenseNet-201) are fixed, and only the succeeding networks are trained, the computational burden is significantly reduced. Thirty-two films, along with their amplified data, are used in each training batch. We fine-tune our model using the Adam method with a learning rate of 0.001. Table 1 shows the model’s hyperparameters.

**Table 1:** Hyperparameter Value.

Hyperparameter	Value
Optimizer	Adam
Loss Function	Categorical Cross-Entropy
Batch Size	32
Number of epochs	Dynamic
Learning Rate	.001
Input Size	40 X 1536 (Extracted Feature set from DenseNet-201)
DropOut	.5
Recurrent Dropout	.5

### 4.3. Performance metrics

The performance metrics used in this work are loss, accuracy, confusion matrix, and accuracy class-wise.

**Loss:** Loss is a quantitative assessment of the degree to which a model’s predictions align with the actual values.

**Confusion matrix:** A confusion matrix is a grid representation that shows the effectiveness of a proposed model by taking the ratio between the predicted classes and the actual classes. The data set comprises four essential metrics: True Positives (TP), True Negatives (TN), False Positives (FP), and False Negatives (FN).

**Accuracy:** Accuracy is the ratio of accurate predictions produced by the model to the total number of predictions and is calculated as equation (8).


$$\begin{array}{c} Accuracy=\frac{Number\;of\;Correct\;Predictions}{/Total\;Number\;of\;Predictions} \end{array}$$ (8)

**Class-wise Accuracy:** Class-wise accuracy refers to the accuracy that is determined for each class. It quantifies the model’s ability to properly forecast occurrences of a certain class, offering insights into its performance across several classes. It is calculated using equation (9).


$$\begin{array}{c} Class-wise\;Accuracy=\frac{Correct\;Predictions\;for\;a\;class}{/Total\;Instances\;of\;that\;class\;} \end{array}$$ (9)

### 4.4. Result

**Fig 3:**
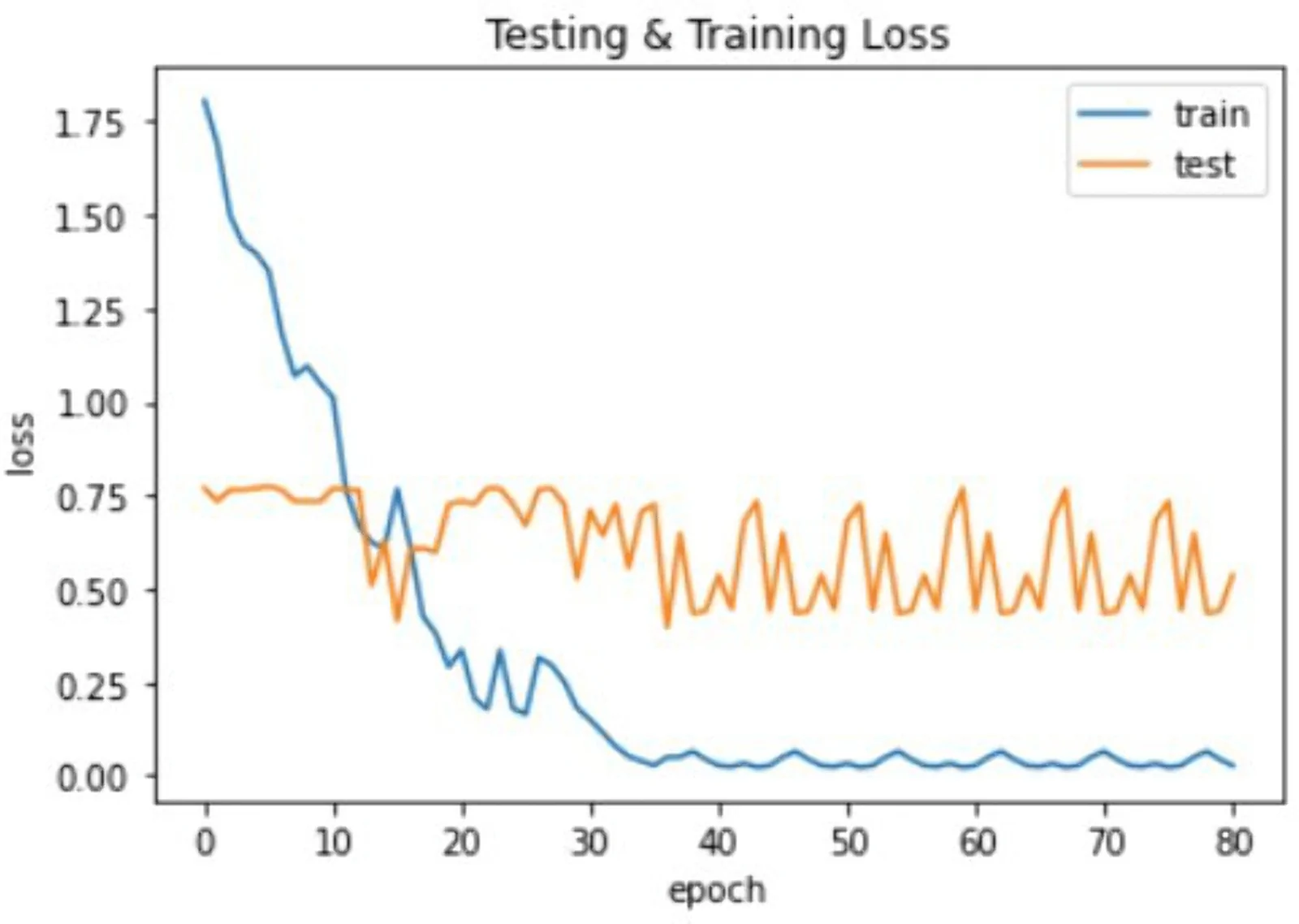
Train and test loss of UCF–crime.

**Fig 4:**
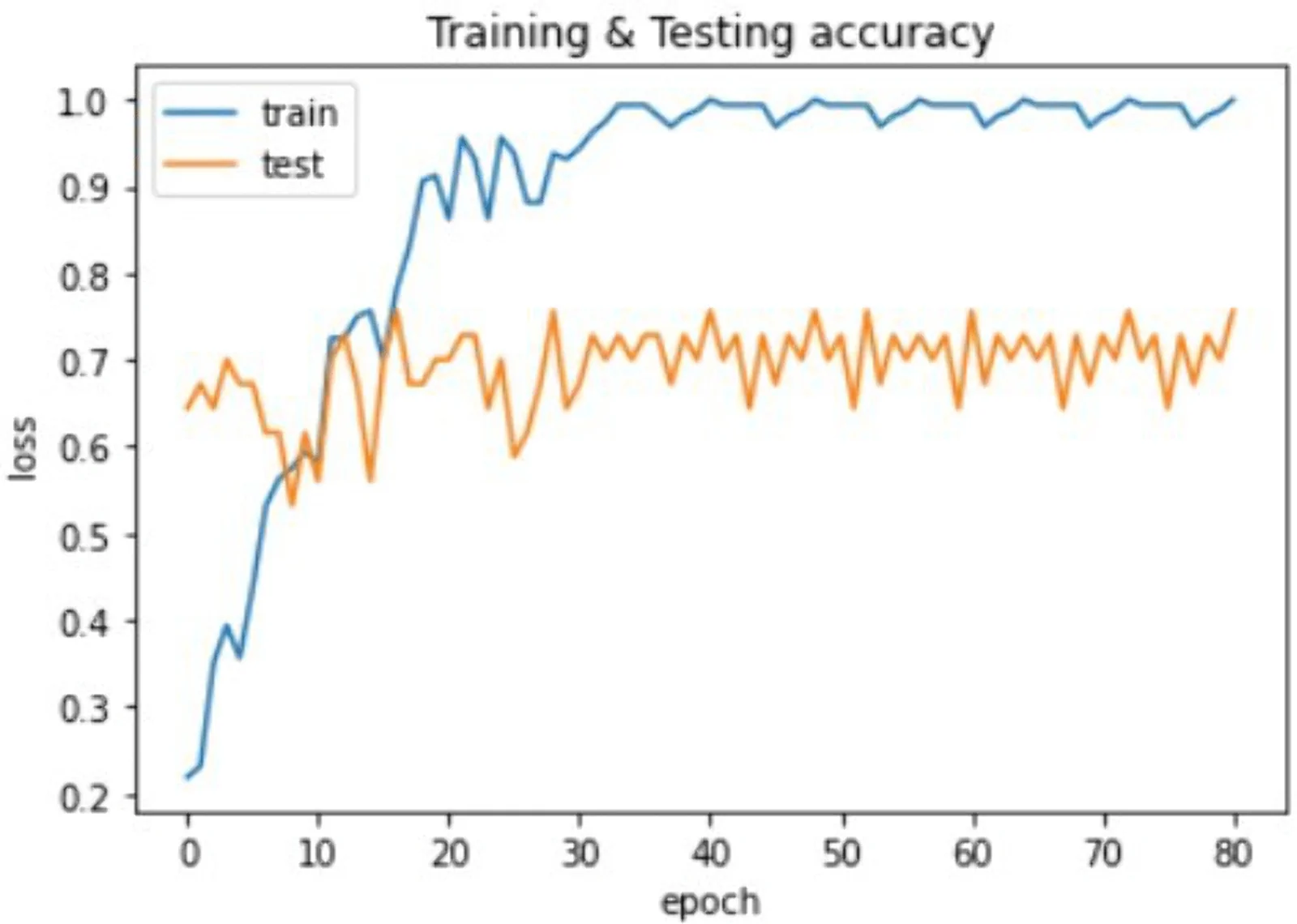
Train and test accuracy of UCF–crime.

**Fig 5:**
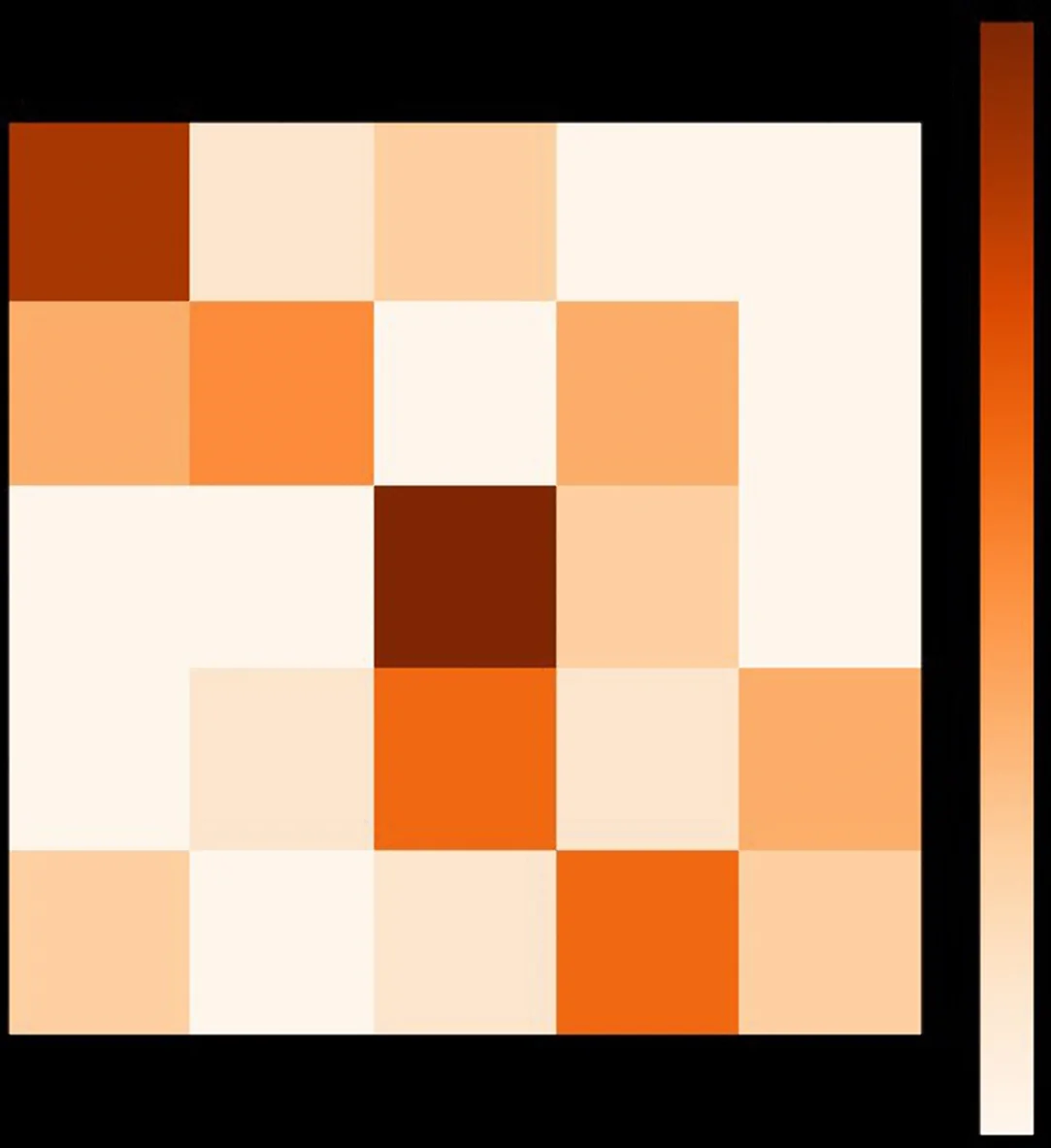
Confusion matrix of UCF–crime.

**Fig 6:**
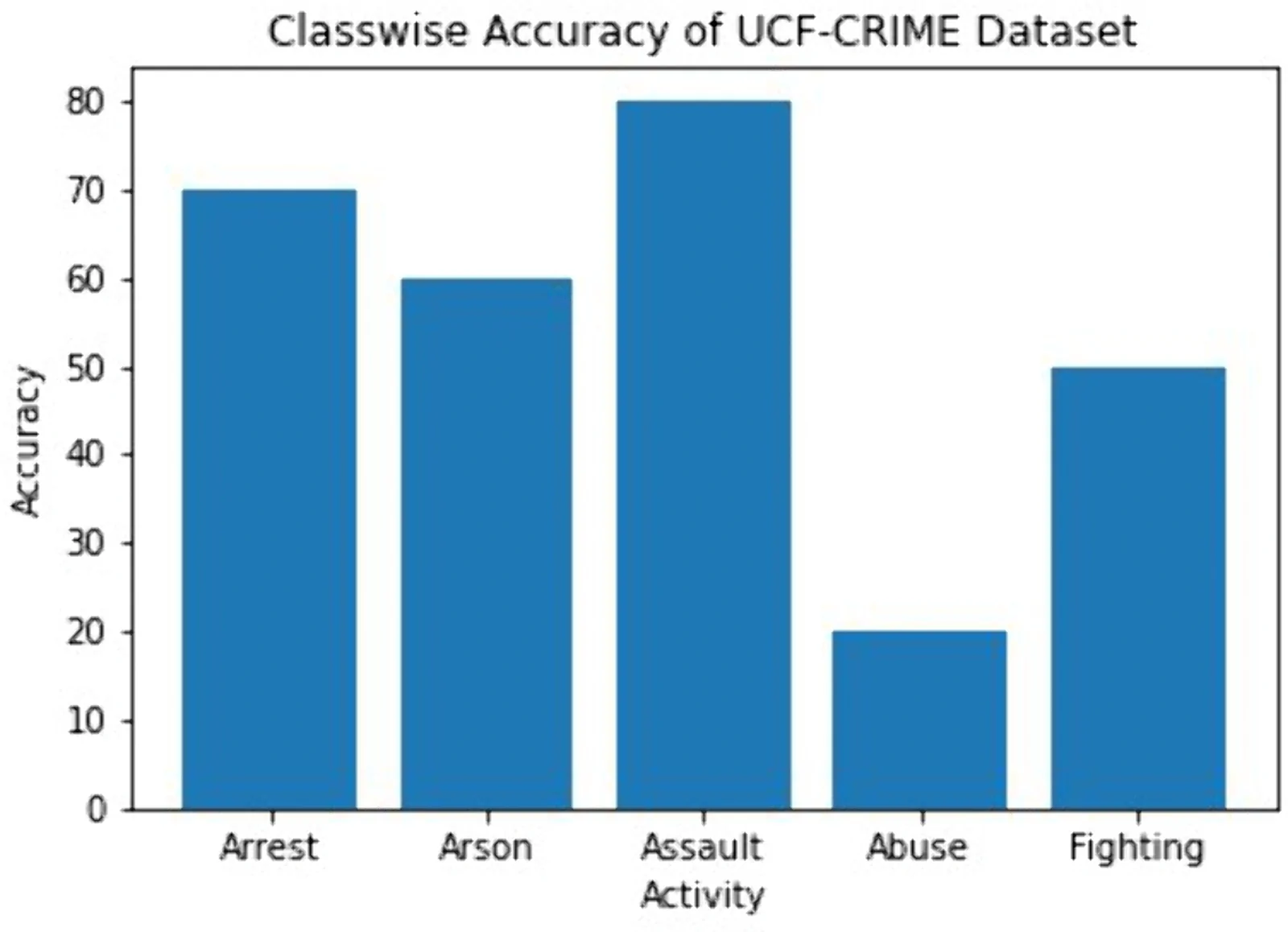
Class –wise accuracy of UCF–crime.

**Result discussion of UCF-Crime:** Fig 3 illustrates that the training loss decreases gradually, whereas the testing loss stabilizes with minor fluctuations after a certain point on the UCF-Crime dataset. Fig 4 illustrates that the accuracy of training improves consistently, while the accuracy of tests stabilizes with minor fluctuations, indicating decent generalization but not flawless generalization. Fig 5 presents the classification performance by the confusion matrix, which emphasizes the areas of correct predictions and misclassifications for various classes in UCF-Crime. Fig 6 illustrates the class-wise accuracy of the UCF-Crime categories, with Assault Activity obtaining the highest accuracy and abuse the lowest.

Each video contains over 2300 frames. The proposed framework is compared against five human activity detection frameworks, which are listed in Table 2. The table contrasts the performance to identify anomalies in the UCF-Crime dataset. I3D and C3D CNN SIAMESE [40] achieve relatively low accuracies of 28.8% and 31.5%, respectively. The accuracy of the Discriminative Anomalous Clip Miner methods [41], both with and without Non-Local (NL) modules, is enhanced to 34.1% and 35.1%, respectively. The proposed method is substantially more effective than others, as accuracy is 62.04%.

**Table 2:** Accuracy on UCF-crime dataset.

S.No	Method	Accuracy %
1.	I3D CNN SIAMESE MethodMethod	28.8
2.	C3D CNN SIAMESE	31.5
3.	Discriminative Anomalous Clip Miner (without NL)	34.1
4.	Discriminative Anomalous Clip Miner ( NL)	35.1
5.	CNN+LSTM	44.67
6.	Our Proposed	62.04

**Result discussion of UCF50:** The UCF50 dataset is utilized to contrast the proposed method with five human activity recognition methods, including Bag of Expression [42], VGG-19 + LinearSVM [43], VGG-19 + QuadSVM [43], ConvLSTM, and LRCN [44].

The proposed method outperforms Bag of Expression, VGG-19 with linear and quadSVM, convLSTM, and LRCN for the UCF50 dataset, as illustrated in Table 3. The proposed procedure has an accuracy of 93.42%, 92.6%, 94.9%, 81.97%, 93.44%, and 94.9%, respectively. In comparison to the most recent accuracy result from LRCN to Hierarchical clustering multi-task models, the proposed method enhances accuracy by.14% and 13.03%, respectively.

**Table 3:** Accuracy of the presented Model on the UCF 50 dataset.

S. No	𝐌𝐞𝐭𝐡𝐨𝐝	𝐀𝐜𝐜𝐮𝐫𝐚𝐜𝐲 %
1.	Bag of Expression (2018)	93.42
2.	VGG−19 + LinearSVM (2021)	92.6
3.	VGG−19 + QuadSVM (2021)	94.9
4.	ConvLSTM(2022)	81.97
5.	LRCN(LongTerm Recurrent Convolutional Network)(2022)	93.44
6.	𝐎𝐮𝐫 𝐏𝐫𝐨𝐩𝐨𝐬𝐞𝐝	95.04

**Fig 7:**
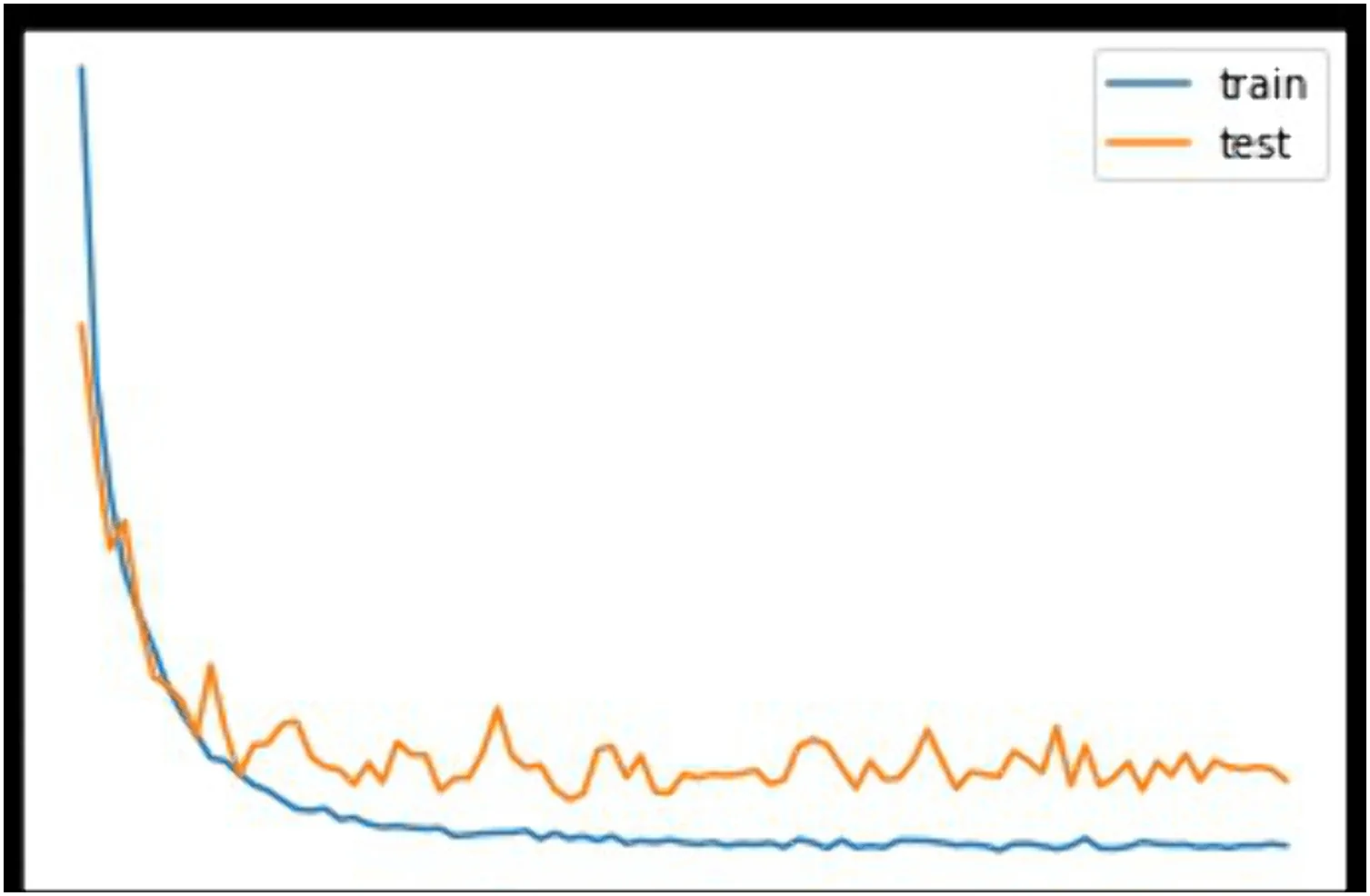
Test and train loss of UCF50.

**Fig 8:**
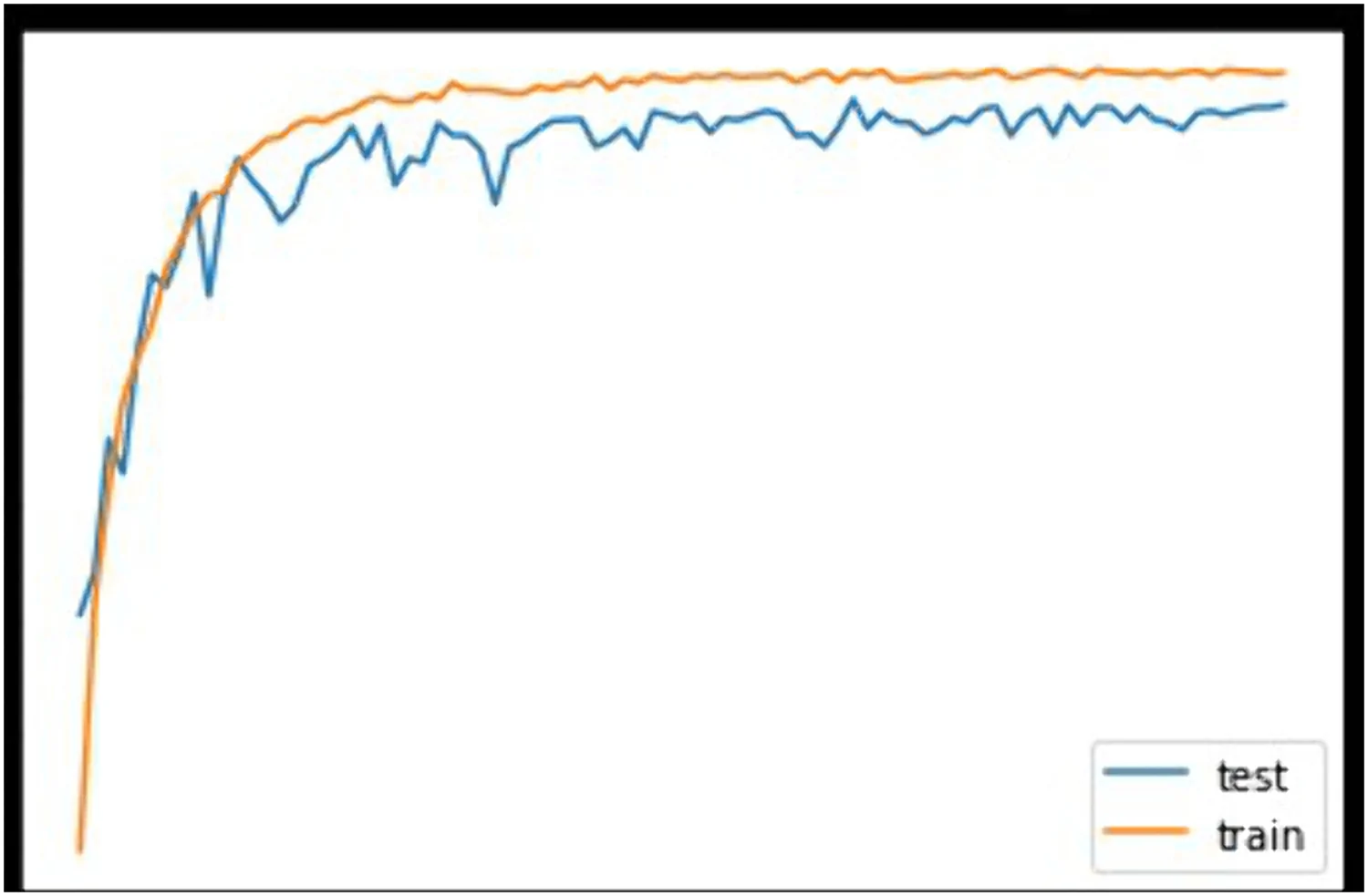
Test and train accuracy of UCF50.

**Fig 9:**
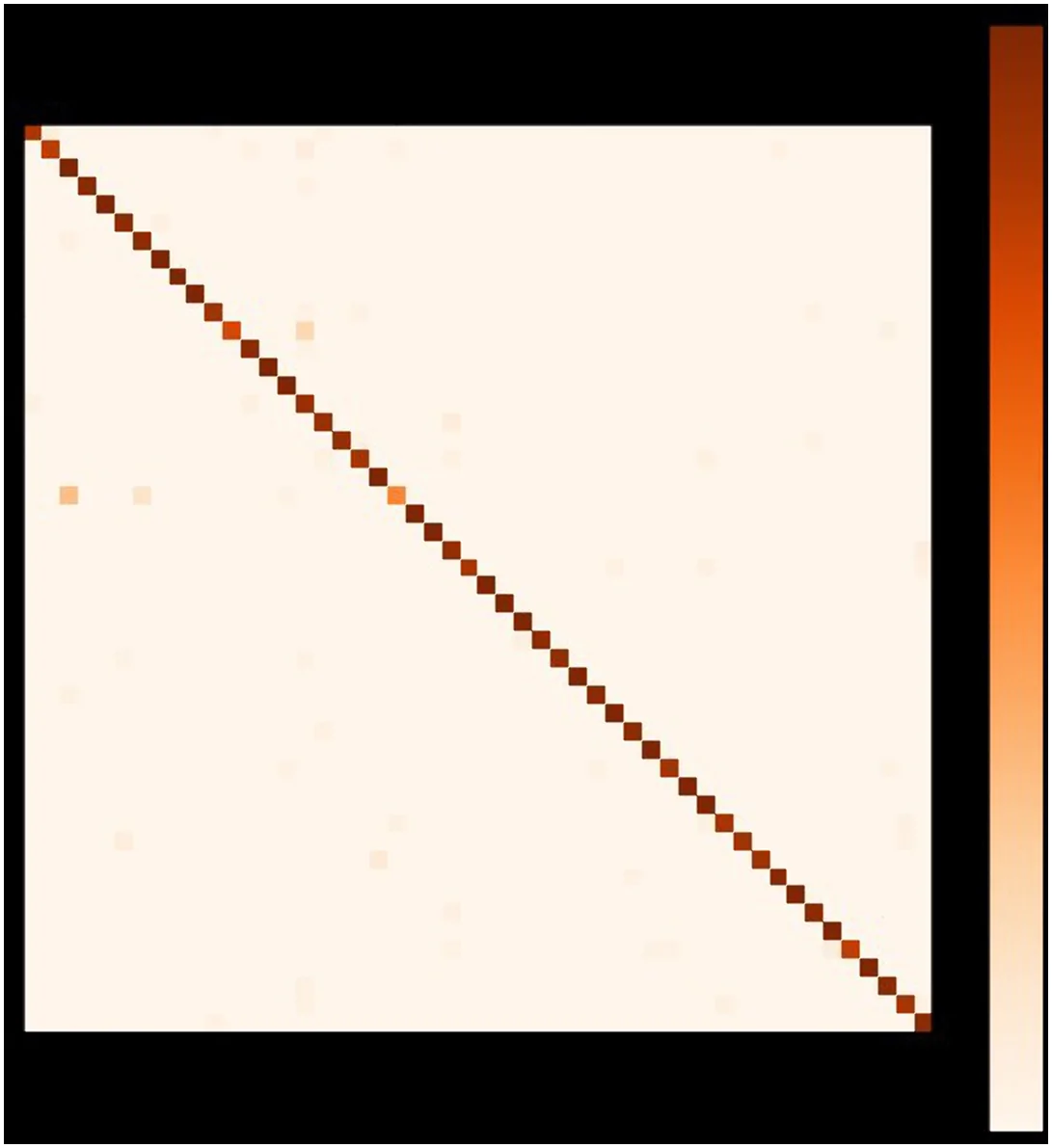
Confusion matrix of UCF50.

**Fig 10:**
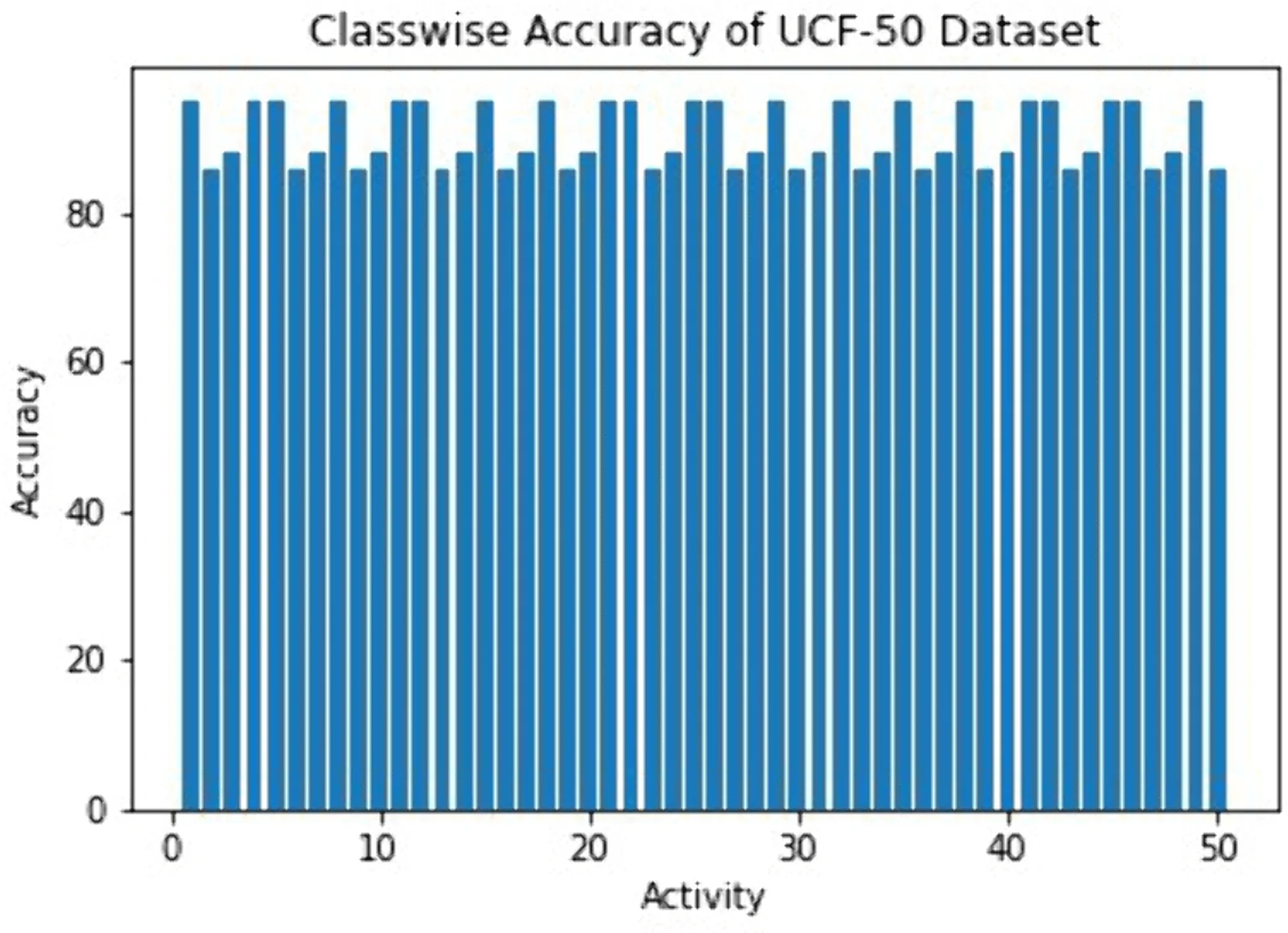
Class–wise accuracy of UCF50.

Fig 7 illustrates the variation in training loss as well as testing loss, while Fig 8 highlights the training and testing accuracies. Fig 9 shows the confusion matrix, which illustrates that the model is stable. Fig 10 presents class-wise accuracies, where almost all activities achieve more than 94% accuracy.

### 4.5. Ablation study

To assess the role that each part plays in the suggested framework, a step-wise ablation study was conducted by sequentially removing or modifying the main components and determining their impact on the performance of activity recognition tasks. They tested three major variants: (i) they replaced DenseNet-201 with a baseline CNN to study whether dense connectivity had a significant effect on spatial representation, (ii) they replaced the Bi-LSTM with the unidirectional LSTM to study whether or not the idea of bidirectional temporal modeling was important, and (iii) they did not use the chunk-based processing to study whether or not the system could process real-time sequential dependencies. A comparison of these variants to the full model is given in Table 4 on the UCF-Crime and UCF-50 datasets. As we saw in the table, all the changes result in a steady drop in performance, indicating that spatial richness, temporal completeness, temporal continuity, and pre-trained knowledge are all essential to effective anomaly detection. When using dense feature propagation and reuse as an alternative to a conventional CNN, the greatest performance decline is noted, which proves that the application of dense feature propagation and reuse is a key to detecting visually subtle irregularities. On the same note, bidirectionality of LSTM is eliminated, and the modeling of the long-range dependencies, as well as preserving the contextual information of the subsequent frames, is compromised. Chunk processing removes temporal smoothness between neighboring frame blocks, which adversely influences prediction stability in real-time streams. Altogether, the findings of the ablation point to the fact that all components have significant contributions to the increased performance of the offered spatiotemporal model.

**Table 4:** Comparison with different variants.

Model Variant	Spatial Module	Temporal Module	Chunk-Based Processing	Transfer Learning	UCF-Crime Accuracy (%)	UCF-50 Accuracy (%)
Full Proposed Model	**DenseNet-201**	**Bi-LSTM**	**✓**	**✓**	***62.04***	***95.04***
Variant-1	Baseline CNN	Bi-LSTM	✓	✓	45.04	87.67
Variant-2	DenseNet-201	Uni-LSTM	✓	✓	60.62	94.67
Variant-3	DenseNet-201	Bi-LSTM (without chunk)	✓	✓	59.62	93.67

**Computational Time:** We have calculated the time complexity of the proposed methodology and presented it in Table 5 in terms of FLOPs, GPU inference time per frame, and the latency introduced by sequential processing. Table 5 indicates the computational advantage of the proposed chunk-based temporal modeling strategy explicitly. Running the complete sequence using full-length Bi-LSTM results in high memory usage and extremely large FLOPs, hence it cannot be deployed in real-time. A 200-frame non-chunking sequence length continues to lead to significant computational overhead and latency. Oppositely, the suggested chunk-based Bi-LSTM is much less memory-consuming and floating-point intensive by constraining the temporal window to 20 frames and thus provides low-latency inference, 38 ms per chunk. The result of this limited temporal complexity is stable memory demands and real-time processing of surveillance videos, including long-duration videos.

**Table 5:** Computational Time with Bi-LSTM.

Full-Sequence Bi-LSTM	~2000 frames	6.8 GB	~ 1.2 × 10¹¹	3.4 s/ video	✗ No
Bi-LSTM (No Chunking)	200 frames	2.1 GB	~ 1.9 × 10^10^	820 ms	✗ No
Proposed Chunk-Based Bi-LSTM	20 frames	0.62 GB	~ 2.1 × 10⁹	38 ms/chunk	✓ Yes

## 5. Conclusion

In this work, we have designed a deep framework for the detection of unusual activities in real-time video surveillance using DenseNet-201 and Bi-LSTM. DenseNet-201 extracted the high-level features using its densely connected layers, while Bi-LSTM processed the feature vector in both directions, capturing the temporal dependencies among frames. We have used chunk-based processing to handle the unavailability of future frames in real-time video surveillance. Our proposed framework performed better compared to leading methods with an accuracy 62.01% on the UCF crime and 95.04% on the UCF 50 dataset. Besides the detection performance, we have discussed the interpretability aspects of the proposed spatiotemporal framework. The hierarchical spatial codes of DenseNet-201 and the forward and backward temporal codes of Bi-LSTM permit the activation patterns and temporal change of anomaly analysis, which confirm transparent and explainable decision-making in the surveillance environment. For handling the future frame in real-world surveillance, we will use an improved scheme, which reduces the latency time.
